# A case–control study of occupation/industry and renal cell carcinoma risk

**DOI:** 10.1186/1471-2407-12-344

**Published:** 2012-08-08

**Authors:** Sara Karami, Joanne S Colt, Kendra Schwartz, Faith G Davis, Julie J Ruterbusch, Stella S Munuo, Sholom Wacholder, Patricia A Stewart, Barry I Graubard, Nathanial Rothman, Wong-Ho Chow, Mark P Purdue

**Affiliations:** 1 Division of Cancer Epidemiology and Genetics, National Cancer Institute, NIH, DHHS, 6120 Executive Boulevard, MSC 7242, Bethesda, MD 20892-7242, USA; 2 Wayne State University, Karmanos Cancer Institute, 110 E. Warren, Detroit, MI, 48201, USA; 3 University of Illinois, 877 SPHPI M/C 923, 1603 W. Taylor Street, Chicago, IL, 60612, USA; 4 Formerly of Information Management Service Inc, 6110 Executive Blvd Suite #310, Rockville, MD, 20852, USA; 5 Stewart Exposure Assessments, LLC, Arlington, VA, USA; 6 Formerly of the Division of Cancer Epidemiology and Genetics, National Cancer Institute, NIH, DHHS, Bethesda, MD, USA

**Keywords:** Kidney cancer, Renal cancer, Clear cell RCC, Occupation, Industry, Race

## Abstract

**Background:**

The role of occupation in the etiology of renal cell carcinoma (RCC) is unclear. Here, we investigated associations between employment in specific occupations and industries and RCC, and its most common histologic subtype, clear cell RCC (ccRCC).

**Methods:**

Between 2002 and 2007, a population-based case–control study of Caucasians and African Americans (1,217 cases; 1,235 controls) was conducted within the Detroit and Chicago metropolitan areas to investigate risk factors for RCC. As part of this study, occupational histories were ascertained through in-person interviews. We computed odds ratios (ORs) and 95% confidence intervals (CIs) relating occupation and industry to RCC risk using adjusted unconditional logistic regression models.

**Results:**

Employment in the agricultural crop production industry for five years or more was associated with RCC (OR = 3.3 [95% CI = 1.0-11.5]) and ccRCC in particular (OR = 6.3 [95% CI = 1.7-23.3], *P* for trend with duration of employment = 0.0050). Similarly, RCC risk was elevated for employment of five years or longer in non-managerial agricultural and related occupations (OR_RCC_ = 2.1 [95% CI = 1.0-4.5]; OR_ccRCC_ = 3.1 [95% CI = 1.4-6.8]). Employment in the dry-cleaning industry was also associated with elevated risk (OR_RCC_ = 2.0 [95% CI = 0.9-4.4], *P* for trend = 0.093; OR_ccRCC_ = 3.0 [95% CI = 1.2-7.4], *P* for trend = 0.031). Suggestive elevated associations were observed for police/public safety workers, health care workers and technicians, and employment in the electronics, auto repair, and cleaning/janitorial services industries; protective associations were suggested for many white-collar jobs including computer science and administrative occupations as well employment in the business, legislative, and education industries.

**Conclusions:**

Our findings provide support for an elevated risk of RCC in the agricultural and dry-cleaning industries and suggest that these associations may be stronger for the ccRCC subtype. Additional studies are needed to confirm these findings.

## Background

Malignant tumors of the kidney account for about 2% of cancer diagnoses worldwide [1]. In the United States (U.S.), kidney cancer accounts for approximately 4% of newly diagnosed cancer cases and 2% of cancer deaths [2,3]. The most common form, renal cell carcinoma (RCC) of the renal parenchyma, accounts for more than 85% of kidney cancers [3,4]. RCC includes several histologic subtypes, the most common of which is clear cell RCC (ccRCC), making up approximately 70% of cases [5]. These subtypes possess different genetic, clinical, and demographic characteristics [5,6]; differences in etiology have also been speculated [7].

The etiology of RCC is complex and not well understood. Cigarette smoking, excess body weight and hypertension are well established risk factors that account for nearly half of all RCC diagnoses in the U.S. [1,3,4]. Although generally not considered an occupational disease, an association between RCC and occupational risk factors has been suggested in a number of epidemiological studies [4,8-16]. Most recently, a large Eastern European case–control study reported statistically significant increased RCC risk for workers in the agricultural and animal husbandry industries, particularly among farmers [8]. Other industries and occupations that have been linked to RCC risk, although not consistently, include printers [3,9-11], mechanics and repairers [3,9,12], metal workers [3,8-11,13], truck drivers [13,14], railroad workers [3,10,11,13], aircraft mechanics [3,9], and those employed in the dry-cleaning [9,15], petroleum [10,13,15], iron and steel [4,13,16], and printing [9,10,13] industries. To our knowledge, no studies of occupation and RCC have investigated associations with RCC subtypes.

To further explore the relationship between occupation and RCC risk, we analyzed lifetime occupational histories collected from participants of a population-based case–control study of Caucasians and African Americans conducted in the U.S. The study was designed to explore a variety of risk factors in the etiology of RCC, and to examine whether the risk factors varied by race.

## Methods

### Study population

Caucasian and African American male and female residents of Chicago, Illinois (Cook County) and Detroit, Michigan (Macomb, Oakland, and Wayne Counties) were the source population for this study. All incident cases of histologically confirmed adenocarcinoma of the kidney (ICD-O C64) between 20 and 79 years of age diagnosed within the enrollment periods for Chicago (January 1, 2003 through December 31, 2003) and Detroit (February 1, 2002 through January 31, 2007 for African Americans and through July 31, 2006 for Caucasians) were eligible to participate. Potential cases from the Detroit area were identified through the Metropolitan Detroit Cancer Surveillance System, a cancer registry of the National Cancer Institute’s Surveillance, Epidemiology, and End Results (SEER) program. In Chicago, potential cases were identified from pathology reports issued at participating hospitals in Cook County and adjacent communities. Controls were recruited from the general population, with frequency matching to the case series on the basis of age group, self-reported race, sex, and study center. Controls aged 65–79 years were identified from files of the Centers for Medicare and Medicaid Services, and controls under age 65 years were identified from Department of Motor Vehicle (DMV) records.

Although RCC rates are higher among African Americans than Caucasians [3], many more Caucasian than African American RCC cases were diagnosed in the study areas, as expected given the greater number of Caucasian residents. This limited power for analysis of risk factors by race. Therefore, a sampling strategy was designed to recruit a sufficient number of African Americans efficiently [17], that is, without exceeding recruitment goals for Caucasians. All African American cases were recruited, while some age-sex strata of Caucasian cases were subsampled. To further increase power for analyses restricted to African Americans, the study maintained a control:case matching ratio of 2:1 for African Americans. For Caucasians, with larger numbers of cases, there was less need for additional power, and we therefore matched at a ratio of 1:1. Information on race was unavailable from DMV records, hampering our ability to frequency match controls to the cases among those 20 to 64 years of age. Therefore, we used the racial density of the census block group (according to the U.S. 2000 Census) in which each control resided as a surrogate for race for the purposes of sampling, and over-sampled people living in high-density African American areas [17].

Of the 1,918 eligible cases identified, 171 died prior to contact or interview, 92 could not be located with the available contact information, 21 moved out of the area, and the physicians of 63 cases refused permission to contact their patients. Among the remaining 1,571 cases we sought to enroll, 221 declined participation and 133 were not interviewed due to serious illness, impairment, or not responding to multiple attempts to contact. Thus, 1,217 cases (77.5% of those we attempted to recruit) participated in the study. Of the 2,718 presumed eligible controls, 41 died prior to contact or interview, 345 could not be located with the available contact information, and 63 had moved out of the region. Among the 2,269 controls we attempted to recruit, 677 declined to participate and 357 were not interviewed due to serious illness, impairment, or not responding to multiple attempts to contact. Thus, 1,235 eligible controls (54.4% of those we attempted to recruit) participated. Approvals were obtained from human subjects review boards at all participating institutions, and informed written consent was obtained from all participants.

Copies of medical records were obtained from all cases to confirm diagnosis and collect information on histologic and clinical factors. In addition, the original diagnostic slides were obtained for 706 cases for review by an experienced pathologist. We assigned histology on the basis of the centralized histopathologic review if available; otherwise, information from the original diagnostic pathology reports was used.

### Data collection

Those who agreed to participate were scheduled for an in-home, computer-assisted personal interview. Prior to the interview, a work history calendar was mailed to the home, and participants were asked to record information on job title, tasks performed, equipment and chemicals regularly used, and years employment began and ended, for all jobs that were held for at least 12 months. Trained interviewers reviewed the work history calendars at the time of the interview to ensure that the data was complete as they entered the information into the computer. Other information collected during the in-home interview included data on demographics, smoking history, medical and medication history, diet, and family history of cancer.

### Occupational coding and statistical analysis

The Standard Occupational Classification (SOC) [18] and Standard Industry Classification (SIC) [19] schemes were used to code each job held by each participant. RCC and ccRCC risk was estimated for ever/never employment and by duration of employment (never [referent], <5 years, ≥5 years), for every two-, three-, and four-digit SIC and SOC code. Results are presented in the tables only for occupations and industries held by at least 10 study participants. Additional file 1: Table S1 and Additional file 2: Table S2 provide results for all subjects combined, and separately by sex and race, for every industry and occupation reported.

Results are presented first for occupations and industries suspected *a priori* to be associated with RCC risk. *A priori* jobs were determined by reviewing the literature and identifying all occupations or industries significantly (*P*-value ≤0.05) associated with kidney cancer risk in at least two published studies. All studies written in English, and identified in PubMed using the keywords *kidney cancer and occupation*, *kidney cancer and industry*, or *kidney cancer and jobs,* were examined. Results for *a posteriori* high- and low-risk occupations and industries as well as for ccRCC are presented only if we observed (1) a significant association with ever employment or (2) both a significant association with duration of employment and, to increase the likelihood of capturing duration-response relationships that were monotonic in nature, a *P*-value for ever employment of 0.10 or lower.

For analytic purposes, a set of sample weights was developed to reduce the potential for bias arising from differential sampling rates for controls and cases, from survey nonresponse, and from deficiencies in the coverage of the population at risk by the files of the DMV and Centers for Medicare and Medicaid Services to select the controls. Sample weights also include a poststratification adjustment so that the weighted distribution of controls across the matching variables matched exactly the weighted distribution of cases. In addition to being consistent with the objectives of the frequency matching, this poststratification adjustment reduces the variability of the weights [20]. Full detail of the development of the sample weights has been described previously [17].

The sample-weighted frequency distributions of selected characteristics and known RCC risk factors were compared between cases and controls using a Wald F-test [21]. Unconditional logistic regression models using poststratified weights were used to calculate ORs and 95% CIs associated with work history and duration of employment, using individuals never employed in the occupation or industry as the reference group. Tests for trend were performed by modeling medians of employment duration as an ordinal variable and applying the Wald Chi-Square test [21]. The jackknife replicate weight method was used to estimate standard errors [22]. Regression models were adjusted for RCC risk factors which included self-reported hypertension history (ever, never), smoking status (never, occasional, former, current), BMI (self-reported height and weight five years prior to interview) as well as sex, age (at diagnosis for cases and at study selection for controls), race, and family history of cancer. Regression models were additionally adjusted for study center and level of education given that an individual’s work environment and potential occupational exposures is related to these factors. Because previously published studies have shown a link between hypertension and certain occupational exposures (i.e., lead and cadmium) [23,24], analyses were also assessed excluding hypertension from the model; however, no new significant associations were observed. Unweighted unconditional logistic regression analyses for ever employment and duration of employment were also conducted; results were similar to those of the weighted analyses [results not shown]. All analyses were conducted with SAS version 9.2 [SAS Institute, Cary, NC, USA] using procedures appropriate for sample weighted data. Statistical tests were determined to be significant at a two-sided *P*-value <0.05.

## Results

Cases and controls were comparable in sex and age distributions (Table1). Cases were more likely than controls to have a lower education level (*P* <0.001), be current smokers (*P* = 0.03), have a history of hypertension (*P* <0.001), and have excess body weight (body mass index (BMI) >30 kg/m^2^) (*P* <0.001).

**Table 1:** Characteristics of USRCC Cases and Controls

	**Weighted distribution**			
	**Cases**		**Controls**	
**Variables**	**N**	**%**^**a,b**^	**N**	**%**^**a,b**^
**Total**	1,217		1,235	
**Race**				
Caucasian	856	73.9	712	73.9
African American	361	26.1	523	26.1
**Sex**				
Males	720	61.8	689	61.4
Females	497	38.2	546	38.6
**Age at Reference Date**				
<45	147	10.5	179	10.5
45-54	287	21.6	270	21.6
55-64	372	29.4	350	29.4
65-74	303	27.1	329	27.1
75+	108	11.5	107	11.5
**Mean Age**	59.9 years		59.9 years	
**Study Center**				
Detroit	1,018	83.3	1,038	82.7
Chicago	199	16.7	197	17.3
**Education Level**				
<12 years	200	16.7	165	12.0
High School Graduate	419	34.5	390	31.5
Some College	328	26.3	356	27.3
College Graduate	270	22.5	324	29.3
**Smoking Status**				
Never	432	35.3	471	38.4
Occasional ^c^	55	4.7	55	4.0
Regular Former Smoker	410	34.7	445	38.0
Regular Current Smoker	320	25.3	264	19.7
**History of Hypertension**				
No	500	40.8	718	59.0
Yes	701	59.2	508	41.0
**BMI (kg/m**^**2**^**)**^d^				
<25	240	19.5	366	29.1
25-29.9	436	37.4	493	41.7
30-34.99	298	24.9	221	18.3
35+	230	18.2	147	10.9
**Histologic RCC Subtype**				
Clear Cell	709	58.3		
Papillary	169	13.9		
Chromophobe	58	4.8		
other/NOS	281	23.1		

^a^ Due to rounding error, some categories do not sum to 100%. ^b^ A sample weighted frequency distribution. ^c^ Smoked 100 cigarettes in the lifetime, but never smoked >1 cigarette a day for >6 months. ^d^ BMI five years prior to interview. The following data are unknown: BMI (13 cases, 8 controls), history of hypertension (16 cases, 9 controls).

RCC risk associations for all occupations and industries chosen *a priori* are shown in Table2. There were no occupations or industries for which RCC risk increased significantly as duration of employment increased, although a trend of borderline significance was observed for the agricultural crop production industry (Standard Industry Classification (SIC) 01: *P* for trend = 0.051), with a three-fold elevated risk among individuals employed for five or more years (odds ratio (OR) = 3.3 [95% confidence interval (CI) = 1.0-11.5]). Patterns in this industry were similar for men and women ( Additional file 1: Table S1). Significantly increased RCC risk for employment of five years or longer was also observed for agricultural and related occupations, excluding farm managers and proprietors (Standard Occupational Classification (SOC) 56: OR = 2.1 [95% CI = 1.0-4.5], *P* for trend = 0.094), and in particular for non-managerial farm occupations (SOC 561: OR = 3.2 [95% CI = 1.0-10.1], *P* for trend = 0.060). There was a suggestion of elevated risk in the dry-cleaning plant industry (SIC 7216: OR for ever employed = 2.0 [95% CI = 0.9-4.4], increasing to OR = 2.5 [95% CI = 0.4-14.4] for employment of ≥5 years (*P* for trend = 0.093)), particularly among men ( Additional file 1: Table S1). We observed a significant reduction in risk with increasing duration of employment as a mechanic or repairer (SOC 61: *P* for trend = 0.038). Stratified analyses by race or sex showed no noteworthy differences in association for other *a priori* jobs ( Additional file 1: Table S1 and Additional file 2: Table S2).

**Table 2:** **Risk of renal cell carcinoma for*a priori*occupations and industries**

**Job Code & Description**	**NEVER**		**EVER**		**< 5 YEARS**		**5+ YEARS**							
	**Case/Control**		**Case/Control**	**OR**^**a**^**[95% CI]**	**Case/Control**	**OR**^**a**^**[95% CI]**	**Case/Control**	**OR**^**a**^**[95% CI]**	***P*-trend**					
**OCCUPATION:**														
SOC 11–13: officials and administrators, other	983/1003	1.0	201/208	0.9[0.7-1.2]	38/57	0.7[0.4-1.1]	163/151	1.0[0.8-1.4]	0.81					
SOC 1633: electrical and electronic engineers	1177/1199	1.0	7/12	0.6[0.2-2.0]	1/3	0.2[0.0-1.6E + 09]	6/9	0.8[0.2-3.1]	0.67					
SOC 42: sales occupations, commodities except retail	1122/1161	1.0	62/50	1.4[0.9-2.1]	14/10	1.4[0.6-3.1]	48/40	1.4[0.9-2.1]	0.088					
SOC 43: sales occupations, retail	903/927	1.0	281/284	1.0[0.9-1.2]	186/179	1.1[0.9-1.4]	95/105	0.9[0.7-1.3]	0.76					
SOC 5123: firefighting occupations	1176/1204	1.0	8/7	1.4[0.4-4.7]	3/1	3.2[0.0-8.8E + 09]	5/6	1.1[0.3-4.8]	0.81					
SOC 551: farmers (working proprietors)	1178/1207	1.0	6/4	2.4[0.5-11.3]	5/3	2.4[0.4-15.5]	1/1	2.2[0.0-5.3E + 13]	0.67					
SOC 56: other agricultural & related occupations	1133/1160	1.0	51/51	1.0[0.7-1.6]	28/38	0.7[0.4-1.3]	23/13	2.1[1.0-4.5]*	0.094					
SOC 561: farm occupations, except managerial	1159/1187	1.0	25/24	1.2[0.6-2.1]	14/18	0.7[0.3-1.5]	11/6	3.2[1.0-10.1]*	0.060					
SOC 5612: general farm workers	1180/1205	1.0	4/6	0.9[0.3-2.9]	1/4	0.2[0.0-1.3E + 09]	3/2	3.3[0.4-27.8]	0.36					
SOC 5613: field crop & vegetable farm workers	1176/1202	1.0	8/9	1.0[0.3-3.0]	4/6	0.7[0.1-3.3]	4/3	1.7[0.2-11.2]	0.69					
SOC 562: related agricultural occupations	1158/1183	1.0	26/28	0.9[0.5-1.6]	14/21	0.7[0.3-1.5]	12/7	1.6[0.5-5.0]	0.61					
SOC 61: mechanics and repairers	1065/1081	1.0	119/130	0.8[0.6-1.1]	36/25	1.2[0.7-2.2]	83/105	0.7[0.5-1.0]*	0.038					
SOC 644: painters, paperhangers, and plasterers	1166/1193	1.0	18/18	1.0[0.5-2.1]	9/6	1.0[0.3-3.3]	9/12	1.1[0.4-3.1]	0.88					
SOC 681–682: precision metal workers	1137/1177	1.0	47/34	1.2[0.8-1.9]	14/10	1.4[0.6-3.4]	33/24	1.2[0.7-2.0]	0.48					
SOC 7643: printing machine operators & tenders	1176/1205	1.0	8/6	1.3[0.4-3.9]	6/3	2.1[0.4-10.4]	2/3	0.5[0.0-5.2]	0.71					
SOC 7658: laundering and dry-cleaning machine operators and tenders	1177/1202	1.0	7/9	0.8[0.3-2.3]	4/6	0.6[0.1-2.4]	3/3	1.2[0.2-8.7]	0.997					
SOC 772: assemblers	1043/1085	1.0	141/126	1.1[0.9-1.5]	67/55	1.2[0.8-1.8]	74/71	1.1[0.7-1.6]	0.61					
SOC 821: motor vehicle operators	1030/1085	1.0	154/126	1.2[0.9-1.6]	62/49	1.3[0.9-2.1]	92/77	1.1[0.8-1.6]	0.56					
SOC 8243: sailors and deckhands	1177/1202	1.0	7/9	1.2[0.4-3.4]	3/2	1.7[0.2-17.0]	4/7	1.0[0.3-3.5]	0.93					
**INDUSTRY:**														
SIC 01: agricultural production, crops	1161/1191	1.0	23/20	1.4[0.7-2.8]	13/15	0.9[0.4-2.3]	10/5	3.3[1.0-11.5]	0.051					
SIC 02: agricultural production, livestock	1180/1204	1.0	4/7	0.4[0.1-1.7]	3/5	0.5[0.1-2.6]	1/2	0.3[0.0-1.5E + 09]	0.61					
SIC 29: petroleum and coal productscpe	1177/1207	1.0	7/4	1.6[0.4-6.8]	2/1	2.0[0.0-1.4E + 11]	5/3	1.4[0.3-6.1]	0.59					
SIC 3312: blast furnaces and steel mills	1142/1164	1.0	42/47	0.8[0.5-1.3]	20/17	1.5[0.8-2.9]	22/30	0.5[0.3-1.0]*	0.061					
SIC 7216: dry-cleaning plants, except rug	1169/1198	1.0	15/13	2.0[0.9-4.4]	11/10	1.8[0.6-5.4]	4/3	2.5[0.4-14.4]	0.093					

^a^ Adjusted for sex, age at reference date, race, study center, education level, history of hypertension, smoking status, BMI (5 years prior to interview) and family history of cancer. ^*^P-value <0.05. *P*-trends ≤0.05 for duration (Never, <5 years, 5+ years) of employment are bolded. Results presented if a job was held by ≥10 participants. Occupational results for architects, aircraft mechanics, railway workers, fisherman, sailors, seafarers, petroleum workers, and structural and sheet metal workers not shown due to small numbers. Industry results for blast furnace, coke oven, iron and steel, paper, and petroleum refining not shown due to small numbers.

RCC risks for *a posteriori* high- and low-risk occupations are shown in Table3. Significant elevations in RCC risk, with significant trends with employment duration, were observed for health technologists and technicians (SOC 36: OR = 1.7 [95% CI = 1.1-2.6], *P* for trend = 0.043), pressing machine operators (SOC 7657: OR = 4.7 [95% CI = 1.3-17.4], *P* for trend = 0.044), and machine feeders and off bearers (SOC 8725: OR = 2.2 [95% CI = 1.1-4.3], *P* for trend = 0.024). A nearly five-fold risk was observed among those ever employed as recreational workers (SOC 2033: OR = 4.8 [95% CI = 1.7-13.9]). Stratification by sex and race ( Additional file 2: Table S2) revealed a small number of additional occupations with significant associations for ever employment and significant trends with duration of employment: male insurance workers (SOC 4122: OR = 2.2 [95% CI = 1.1-4.4], *P* for trend = 0.026), female janitors and cleaners (SOC 5244: OR = 2.8 [95% CI = 1.2-6.7], *P* for trend = 0.044), and Caucasian sales workers (SOC 42: OR =1.6 [95% CI = 1.1-2.5], *P* for trend = 0.022). Several statistically significant protective associations were also observed in Table3, mainly for white- collar occupations such as administrators, computer scientists and programmers, librarians, and various administrative support occupations.

**Table 3:** **Risk of renal cell carcinoma for*a posteriori*high- and low-risk occupations**

**Job Code and Description**	**NEVER**		**EVER**		**< 5 YEARS**		**5+ YEARS**		***P-*trend**
	**Case/Control**		**Case/Control**	**OR**^**a**^**[95% CI]**	**Case/Control**	**OR**^**a**^**[95% CI]**	**Case/Control**	**OR**^**a**^**[95% CI]**	
SOC 128: administrators, education & related fields	1179/1192	1.0	5/19	0.3 [0.1-0.8]*	1/3	0.20 [0.0-1.9E + 09]	4/16	0.3 [0.1-0.9]*	**0.037**
SOC 171: computer scientists ^b^	1180/1195	1.0	4/16	0.2 [0.1-0.7]*	1/7	0.1 [0.0-0.7]*	3/9	0.3 [0.1-1.1]	**0.054**
SOC 2033: recreation workers	1175/1206	1.0	9/5	4.8 [1.7-13.9]*	7/5	**---------**	2/0	**---------**	
SOC 25: librarians, archivists, and curators	1182/1203	1.0	2/8	0.2 [0.1-1.0]*	1/3	0.2 [0.0-1.3]	1/5	0.3 [0.0-1.4]	0.096
SOC 26: physicians and dentists	1179/1198	1.0	5/13	0.4 [0.2-0.8]*	1/2	0.5 [0.0-6.8]	4/11	0.4 [0.1-1.1]	0.067
SOC 36: health technologists and technicians	1135/1175	1.0	49/36	1.7 [1.1-2.6]*	19/14	1.7 [0.8-3.8]	30/22	1.6 [1.0-2.7]	**0.043**
SOC 397: programmers	1177/1195	1.0	7/16	0.3 [0.1-0.9]*	1/2	0.3 [0.0-1.0E + 09]	6/14	0.3 [0.1-1.0]*	**0.042**
SOC 403: supervisors: sales occupations, retail	1126/1133	1.0	58/78	0.7 [0.5-1.0]*	15/29	0.4 [0.2-0.7]*	43/49	0.9 [0.5-1.4]	0.36
SOC 46–47: administrative support occupations, including clerical	743/685	1.0	441/526	0.8 [0.6-0.9]*	128/161	0.8 [0.6-1.1]	313/365	0.7 [0.6-0.9]*	**0.0086**
SOC 462: secretaries, stenographers and typists	1064/1062	1.0	120/149	0.7 [0.5-1.0]*	27/49	0.5 [0.3-0.8]*	93/100	0.8 [0.6-1.2]	0.31
SOC 4696: file clerks	1174/1181	1.0	10/30	0.4 [0.2-0.7]*	7/22	0.4 [0.2-0.7]*	3/8	0.4 [0.1-1.4]	0.069
SOC 4715: billing clerks	1177/1193	1.0	7/18	0.4 [0.2-1.0]*	5/12	0.4 [0.1-1.4]	2/6	0.3 [0.1-0.8]*	**0.019**
SOC 474: mail & message distributing occupations ^c^	1141/1146	1.0	43/65	0.7 [0.4-1.1]	22/27	0.8 [0.4-1.6]	21/38	0.5 [0.3-1.0]	**0.050**
SOC 475: material recording, scheduling & distributing clerks	1091/1094	1.0	93/117	0.7 [0.5-0.9]*	49/64	0.7 [0.5-1.1]	44/53	0.6 [0.4-1.0]	**0.033**
SOC 5216: food counter, fountain & related occupations	1165/1178	1.0	19/33	0.5 [0.3-1.0]*	14/28	0.5 [0.3-1.0]*	5/5	0.6 [0.1-3.0]	0.18
SOC 525–526: personal service occupations	1117/1104	1.0	67/107	0.7 [0.5-0.9]*	37/72	0.6 [0.4-0.9]*	30/35	0.8 [0.5-1.4]	0.18
SOC 5263: welfare service aides	1178/1194	1.0	6/17	0.4 [0.2-1.0]*	3/12	0.3 [0.1-1.1]	3/5	0.6 [0.2-2.1]	0.20
SOC 615: electrical & electronic equipment repairers ^d^	1157/1162	1.0	27/49	0.5 [0.3-0.8]*	7/13	0.6 [0.2-1.6]	20/36	0.5 [0.3-0.8]*	**0.0065**
SOC 7657: pressing machine operators	1177/1207	1.0	7/4	4.7 [1.3-17.4]*	5/3	5.1 [0.8-30.5]	2/1	4.1 [1.0-17.8]	**0.044**
SOC 8725: machine feeders and off-bearers	1158/1197	1.0	26/14	2.2 [1.1-4.3]*	16/10	1.9 [0.8-4.1]	10/4	3.2 [1.1-9.3]*	**0.024**

^a^ Adjusted for sex, age at reference date, race, study center, education level, history of hypertension, smoking status, BMI (5 years prior to interview) and family history of cancer. Similar patterns of association were observed for: ^b^ SOC 171 and 1719; ^c^ SOC 474 and 4743; ^d^ SOC 615, 6151, and 6158. *P-value <0.05. *P*-trends <0.05 for duration (Never, <5 years, 5+ years) of employment are bolded. Results presented for *a posteriori* high- and low-risk occupations only if we observed (1) a significant association with ever employment or (2) both a significant association with duration of employment and *P*-value for ever employment of 0.10 or lower. Results presented if a job was held by >10 participants.

Table4 shows RCC risk associations for *a posteriori* high- and low-risk industries. RCC risk was significantly elevated with ever and duration of employment for security/commodity brokers and services (SIC 62: OR = 3.2 [95% CI = 1.2-8.7], *P* for trend = 0.015) and for police protection (SIC 9221: OR = 2.2 [95% CI = 1.0-4.8], *P* for trend = 0.045). Increased RCC association for employment of five years or longer was seen for those in the paper and allied products (SIC 26: OR = 3.3 [95% CI = 1.0-10.9], *P* for trend = 0.046) industry. Notable increased associations without significant duration trends were seen in highway and street construction (SIC 1611: OR = 3.1 [95% CI = 1.0-9.4]), water supply (SIC 4941: OR = 4.3 [95% CI = 1.1-16.2]), hardware/plumbing/heating equipment supply (SIC 507: OR = 6.9 [95% CI = 1.4-33.6]), and home health care services (SIC 8082: OR = 2.8 [95% CI = 1.1-7.2]) industries. Industries with sex-specific associations that were statistically significant for ever employment and had significant trends with duration of employment, other than those mentioned above, were the electronic computers industry (SIC 3571: OR = 0.3 [95% CI = 0.1-1.0], *P* for trend = 0.017) for men, and the electrical and electronics equipment industry (SIC 36: OR = 2.3 [95% CI = 1.0-5.2], *P* for trend = 0.010) and motor vehicle parts and accessories (SIC 3714: OR = 2.1 [95% CI = 1.2-3.8], *P* for trend = 0.014) for women; race-specific analyses did not identify additional industries meeting these criteria ( Additional file 1: Table S1). Inverse associations with RCC risk were observed for those employed in the government, education, computer programming, membership organizations or business service industries.

**Table 4:** **Risk of renal cell carcinoma for*a posteriori*high- and low-risk industries**

**Job Code and Description**	**NEVER**		**EVER**		**< 5 YEARS**		**5+ YEARS**		***P-*trend**
	**Case/Control**		**Case/Control**	**OR**^**a**^**[95% CI]**	**Case/Control**	**OR**^**a**^**[95% CI]**	**Case/Control**	**OR**^**a**^**[95% CI]**	
SIC 1611: highway and street construction	1168/1205	1.0	16/6	3.1 [1.0-9.4]*	8/4	1.9 [0.6-6.4]	8/2	7.4 [0.7-75.7]	0.069
SIC 26: paper and allied products	1162/1196	1.0	22/15	1.8 [0.8-4.0]	12/11	1.3 [0.5-3.6]	10/4	3.3 [1.0-10.9]*	**0.046**
SIC 2711: newspapers	1164/1176	1.0	20/35	0.5 [0.3-0.9]*	14/23	0.6 [0.3-1.2]	6/12	0.3 [0.1-1.0]*	**0.023**
SIC 4941: water supply	1176/1209	1.0	8/2	4.3 [1.1-16.2]*	3/0	**---------**	5/2	**---------**	
SIC 507: hardware, plumbing heating equipment supplies	1173/1210	1.0	11/1	6.9 [1.4-33.6]*	7/0	**---------**	4/1	**---------**	
SIC 5461: retail bakeries	1170/1203	1.0	14/8	2.1 [0.9-4.8]	7/6	1.6 [0.5-4.7]	7/2	3.7 [1.0-14.2]	**0.046**
SIC 62: security, commodity brokers & services	1170/1203	1.0	14/8	3.2 [1.2-8.7]*	5/5	2.0 [0.5-9.2]	9/3	4.6 [1.3-16.2]*	**0.015**
SIC 73: business services ^b^	1049/1033	1.0	135/178	0.7 [0.5-0.9]*	71/91	0.7 [0.5-1.1]	64/87	0.6 [0.4-0.8]*	**0.0012**
SIC 737: computer programming, data processing, other repair	1165/1184	1.0	19/27	0.6 [0.3-1.0]	8/7	1.0 [0.4-2.6]	11/20	0.5 [0.2-1.0]*	**0.038**
SIC 738: miscellaneous business services	1132/1147	1.0	52/64	0.7 [0.5-1.1]	27/42	0.6 [0.4-1.0]*	25/22	0.9 [0.5-1.7]	0.56
SIC 8062: general medical and surgical hospitals	1177/1194	1.0	7/17	0.4 [0.1-0.9]*	0/8	**---------**	7/9	**---------**	
SIC 8069: specialty hospitals, except psychiatric	1181/1196	1.0	3/15	0.2 [0.0-1.0]*	3/8	**---------**	0/7	**---------**	
SIC 8082: home health care services	1171/1202	1.0	13/9	2.8 [1.1-7.2]*	8/4	5.2 [1.5-18.2]*	5/5	1.4 [0.3-6.7]	0.41
SIC 82: educational services ^c^	1020/992	1.0	164/219	0.7 [0.6-0.9]*	77/91	0.8 [0.6-1.1]	87/128	0.6 [0.5-0.8]*	**0.0018**
SIC 8361: residential care	1171/1176	1.0	13/35	0.4 [0.2-0.9]*	5/17	0.4 [0.1-1.0]	8/18	0.5 [0.2-1.4]	0.13
SIC 86: membership organizations	1151/1158	1.0	33/53	0.6 [0.4-1.0]	16/20	0.8 [0.4-1.7]	17/33	0.5 [0.3-1.0]	**0.051**
SIC 91: executive, legislative & general government ^d^	1173/1183	1.0	11/28	0.4 [0.2-0.8]*	7/8	0.8 [0.2-2.9]	4/20	0.2 [0.1-0.7]*	**0.011**
SIC 9221: police protection ^e^	1158/1195	1.0	26/16	2.2 [1.0-4.8]*	3/4	1.3 [0.2-7.4]	23/12	2.4 [1.0-5.7]*	**0.045**
SIC 97: national security & international affairs ^f^	962/966	1.0	222/245	0.8 [0.6-1.0]*	122/135	0.7 [0.6-1.0]*	100/110	0.9 [0.6-1.1]	0.29

^a^ Adjusted for sex, age at reference date, race, study center, education level, history of hypertension, smoking status, BMI (5 years prior to interview) and family history of cancer. Similar patterns of association were observed for: ^b^ SIC 73, 734 and 7349; ^c^ SIC 82 and 8221; ^d^ SIC 91 and 9199; ^e^ SIC 92, 922, 9221; ^f^ SIC 97 and 9711. *P-value <0.05. *P-*trends <0.05 for duration (Never, <5 years, 5+ years) of employment are bolded. Results presented for *a posteriori* high- and low-risk industries only if we observed (1) a significant association with ever employment or (2) both a significant association with duration of employment and *P-*value for ever employment of 0.10 or lower. Results presented if a job was held by >10 participants.

Significant associations between ccRCC and *a priori* and *a posteriori* occupations and industries from above are shown in Table5. Most associations remained essentially unchanged when analyses were restricted to cases with clear cell histologic subtype. However, the OR increased for the agricultural crops industry (SOC 01: OR = 3.0 [95% CI = 1.0-8.9]), and the trend with duration of employment became statistically significant (*P* = 0.0050). Similarly, for agricultural and related occupations, excluding farm managers and proprietors (SOC 56), the OR for employment of five years or longer increased to 3.1 (95% CI = 1.4-6.8), with a now significant trend with duration of employment (*P* = 0.0096); the association was particularly strong for non-managerial farm occupations (SOC 561: OR = 5.9 [95% CI = 1.8-19.0], *P* for trend = 0.0020). Associations also strengthened somewhat for employment in the dry-cleaning plant (SOC 7216: OR = 3.0 [95% CI = 1.2-7.4], *P* for trend = 0.031) and the private household industries (SIC 88: OR = 2.4 [95% CI = 1.3-4.4], *P* for trend = 0.018), and for private household cleaners and servants (SOC 507: OR = 3.5 [95% CI = 1.2-10.2], *P* for trend = 0.029).

**Table 5:** Risk of clear cell renal cell carcinoma for select occupations and industries

**Job Code & Description**	**NEVER**		**EVER**		**< 5 YEARS**		**5+ YEARS**		***P-*trend**
	**Case/Control**		**Case/Control**	**OR**^**a**^**[95% CI]**	**Case/Control**	**OR**^**a**^**[95% CI]**	**Case/Control**	**OR**^**a**^**[95%CI]**	
**OCCUPATION:**									
SOC 171: computer scientists	688/1195	1.0	2/16	0.2[0.0-1.0]*	1/7	0.2[0.0-1.2]	1/9	0.2[0.0-1.7E + 09]	0.67
SOC 36: health technologists and technicians ^b^	656/1175	1.0	34/36	2.0[1.2-3.2]*	13/14	2.1[0.9-4.8]	21/22	1.9[1.0-3.7]*	**0.030**
SOC 4122: insurance sales occupations	674/1195	1.0	16/16	2.2[1.0-4.8]	5/9	1.1[0.4-2.9]	11/7	3.4[1.1-10.1]*	**0.029**
SOC 4242: sales representatives, commercial and industrial equipment/supplies	675/1199	1.0	15/12	2.4[1.1-5.6]*	3/5	1.4[0.3-7.4]	12/7	3.0[1.1-8.2]*	**0.028**
SOC 46–47: administrative support occupations, including clerical	429/685	1.0	261/526	0.8[0.6-0.9]*	81/161	0.8[0.6-1.1]	180/365	0.7[0.6-0.9]*	**0.012**
SOC 4696: file clerks	685/1181	1.0	5/30	0.3[0.2-0.8]*	4/22	0.4[0.1-0.8]*	1/8	0.3[0.1-1.4]	0.059
SOC 475: material recording, scheduling & distributing clerks	639/1094	1.0	51/117	0.6[0.4-0.9]*	27/64	0.6[0.4-1.0]	24/53	0.6[0.4-1.1]	**0.048**
SOC 507: private household cleaners & servants ^c^	681/1201	1.0	9/10	3.5[1.2-10.2]*	5/8	2.7[0.7-10.7]	4/2	6.7[1.0-47.1]	**0.029**
SOC 56: other agricultural and related occupations	655/1160	1.0	35/51	1.2[0.8-2.0]	17/38	0.7[0.3-1.5]	18/13	3.1[1.4-6.8]*	**0.0096**
SOC 561: farm occupations, except managerial	671/1187	1.0	19/24	1.6[0.8-3.2]	9/18	0.8[0.3-1.9]	10/6	5.9[1.8-19.0]*	**0.0020**
SOC 61: mechanics and repairers ^d^	628/1081	1.0	62/130	0.7[0.5-1.0]	20/25	1.1[0.5-2.2]	42/105	0.6[0.4-0.9]*	**0.024**
SOC 687: precision food production occupations	682/1207	1.0	8/4	3.5[1.1-10.8]*	3/1	11.1[0.0-3.5E + 10]	5/3	2.3[0.6-9.1]	0.14
**INDUSTRY:**									
SIC 01: agricultural production, crops	675/1191	1.0	15/20	3.0[1.0-8.9]*	6/15	0.6[0.2-2.1]	9/5	6.3[1.7-23.3]*	**0.0050**
SIC 5461: retail bakeries	680/1203	1.0	10/8	2.4[1.1-5.5]*	5/6	1.8[0.7-5.0]	5/2	4.5[1.0-20.6]	**0.033**
SIC 62: security, commodity brokers and services ^e^	680/1203	1.0	10/8	3.5[1.2-10.0]*	3/5	2.1[0.4-10.5]	7/3	5.2[1.4-19.5]*	**0.013**
SIC 7216: dry-cleaning plants, except rug	677/1198	1.0	13/13	3.0[1.2-7.4]*	10/10	2.9[0.9-9.1]	3/3	3.7[0.6-25.4]	**0.031**
SIC 73: business services ^f^	614/1033	1.0	76/178	0.6[0.5-0.9]*	37/91	0.7[0.4-1.1]	39/87	0.6[0.4-0.9]*	**0.0046**
SIC 737: computer programming, data processing, other repair	679/1184	1.0	11/27	0.5[0.3-1.0]	5/7	1.0[0.3-2.9]	6/20	0.4[0.2-0.9]*	**0.024**
SIC 8069: specialty hospitals, except psychiatric	689/1196	1.0	1/15	0.1[0.0-0.5]*	1/8	**---------**	0/7	**---------**	
SIC 8082: home health care services	682/1202	1.0	8/9	3.2[1.1-9.6]*	5/4	6.7[1.6-28.4]*	3/5	1.4[0.2-8.6]	0.45
SIC 82: educational services ^g^	596/992	1.0	94/219	0.7[0.5-0.9]*	40/91	0.7[0.4-1.0]*	54/128	0.7[0.5-0.9]*	**0.014**
SIC 8361: residential care	684/1176	1.0	6/35	0.4[0.2-0.9]*	4/17	0.6[0.2-1.8]	2/18	0.2[0.0-1.2]	0.055
SIC 88: private households	670/1176	1.0	20/35	2.4[1.3-4.4]*	11/21	2.1[1.0-4.5]	9/14	2.9[1.1-7.7]*	**0.018**
SIC 919: general government, nec	686/1188	1.0	4/23	0.4[0.1-1.0]*	2/5	1.9[0.4-10.0]	2/18	0.2[0.1-0.6]*	**0.0047**
SIC 9221: police protection ^h^	673/1195	1.0	17/16	2.5[1.1-5.8]*	2/4	1.7[0.2-16.0]	15/12	2.6[1.0-6.7]*	**0.039**
SIC 97: national security and international affairs ^i^	569/966	1.0	121/245	0.7[0.6-1.0]*	63/135	0.7[0.5-0.9]*	58/110	0.8[0.6-1.2]	0.35

^a^ Adjusted for sex, age at reference date, race, study center, education level, history of hypertension, smoking status, BMI (5 years prior to interview) and family history of cancer. Similar patterns of association were observed for: ^b^ SOC 36 and 369; ^c^ SOC 50 and 507; ^d^ SOC 615, 6151, and 6158; ^e^ SIC 62 and 621; ^f^ SIC 73 and 734; ^g^ SIC 82 and 8221; ^h^ SIC 922 and 9221; ^i^ SIC 97 and 971. *P-value <0.05. *P-*trends ≤0.05 for duration (Never, <5 years, 5+ years) of employment are bolded. Results presented if a job was held by ≥10 participants. Results presented for occupations and industries associated with RCC risk except for SOC 507 and SIC 88.

## Discussion

Several occupations and industries were associated with significantly elevated RCC risk in this study. Of particular interest are the findings for employment in the agricultural and dry-cleaning industries, both of which have been previously associated with RCC. These associations became stronger when the analysis was restricted to patients with ccRCC, as was the case for private household cleaners and servants.

Increased RCC risk has been reported for agricultural/farming jobs in many [8-12,25-27], but not all [28,29], epidemiologic studies. Elevated risk of RCC was observed for agricultural and animal husbandry workers, dairy and general farmers, field crop and vegetable workers, and farm machinery operators in a large Eastern European multi-center case–control study. The majority of these jobs were also observed to have higher risk associated with longer duration of employment [8]. Among men, a nearly two-fold increase in RCC risk was shown for general farm [9,12] and horticultural [9] workers in two separate population-based case–control studies in Iowa and Canada. Additionally, elevated renal cancer mortality (standardized mortality ratio (SMR) = 2.12) was reported in a cohort study of farmers in Italy [25], while a significant excess in kidney cancer death (proportionate mortality rate = 1.10) was found among Caucasian farmers across 23 U.S. states [26]. Recent updates to the National Cancer Institute’s Agricultural Health Study (AHS) also reported a significantly elevated kidney cancer mortality risk for farmers (relative SMR = 1.62) [27]. However, important evidence to the contrary also showed a significant 18% to 39% reduction in kidney/renal pelvis cancer incidence among AHS farmers and their spouses [28]. Furthermore, an earlier review of cancer patterns among farmers based on reports from 13 studies of varying designs across industrialized countries found a significant 8% reduction (meta-relative risk = 0.92, 95% CI = 0.86-0.98) in kidney cancer risk [29]. While pesticides [8,30-32] have been postulated as the exposure responsible for the elevated RCC risk observed among agricultural workers in some studies, these workers may also be exposed to a variety of other potentially carcinogenic substances, including chlorinated solvents, metals, fertilizers, engine exhaust, animal viruses, and microbes.

Although we did not observe significant associations with RCC risk for the other *a priori* occupations or industries, there was a non-significant doubling of risk in the dry-cleaning industry, an association that strengthened when restricted to patients with clear cell RCC. Previous studies have associated dry-cleaning industry workers with RCC [15,33]; none investigated associations with ccRCC. We also observed non-significant elevations in RCC risk for individuals in the cleaning and janitorial services, paper and allied products, electronics, motor vehicle, and auto repair industries, and with ccRCC for private household cleaners and servants. Researchers have speculated that the increased RCC risk observed for these occupations may be related to solvent exposures [34-37]. In particular, tetrachloroethylene (PCE), the primary solvent used in dry cleaning and frequently handled by metal and petroleum workers, and trichloroethylene (TCE), a chlorinated solvent commonly used as a degreaser in metal and automotive repair industries, have been studied extensively [3,34-38]. Evidence from animal and human studies have shown that exposure to these solvents induces nephrotoxicity and nephrocarcinogenicity [3,36,38,39]; both solvents have been classified by the International Agency for Research on Cancer as “probably carcinogenic to humans” [40]. Some epidemiological studies have linked TCE exposure to somatic mutation of the von Hippel-Lindau (*VHL*) tumor suppressor gene which is thought to result in the development of the majority of ccRCCs [41,42]. These results suggest the nonrandom affinity of mutagenic TCE metabolites for *VHL* may lead to ccRCC, although other epidemiological studies investigating the association between TCE exposure, *VHL* damage, and RCC have not replicated these findings [43,44]. We interpret with caution the finding of elevated RCC risk for some typically solvent-exposed jobs in our study, given that we also observed a significant reduction in risk with increasing duration of employment among mechanics/repairers, an occupation that carries a high probability of solvent exposure.

Other occupations and industries that were significantly associated with elevated RCC and/or ccRCC risk in our study included police and public safety workers, and health care workers and technicians. Non-significant increased renal cancer risk was observed among policemen in three different European cohort studies [10,45,46] and a large RCC case–control study in New Zealand [47]. No association with RCC was observed among male health care workers in a Swedish cohort study [10] or in a population-based RCC case–control study in Denmark [13], although a significant increase for kidney and renal pelvis cancer mortality risk (mortality OR = 1.7) was reported for black participants in a U.S. study of female health care workers [48]. In our study, risk for health care workers and technicians did not vary by race. Given the small number of subjects employed in most of the occupational categories reported above, additional studies are needed to replicate and/or confirm results.

Significantly reduced RCC risk was observed for several white-collar occupations in our study. Although most RCC occupational studies have not reported significant inverse associations for white-collar jobs, Heck and colleagues recently observed a significant 30%-40% reduction in renal cancer risk among Central and Eastern European clerical workers, business workers, and social workers [8]. We know of no mechanism by which these types of jobs may protect against renal cancer risk, and we suspect that these findings could be attributable to a combination of chance and confounding by unknown factors. Additional studies that include white-collar occupations are needed to clarify these possible associations.

Strengths of our study include its population-based design of Caucasians and African Americans, inclusion of only histologically confirmed RCC cancers, and large sample size. Further, our study of RCC is to our knowledge the first to evaluate associations with occupation and industry separately by race, and restricted to cases of clear cell histology. The associations with ccRCC observed in our study for employment in the agricultural and dry-cleaning industries suggest that the biologic effects underlying exposures in these occupations may be particularly relevant to the pathogenesis of this disease subtype. Subtype-specific investigations in other studies are needed to confirm these findings.

Our study also has limitations. Given the large number of occupations and industries evaluated and issues stemming from multiple comparisons, it is likely that some of our findings arose due to chance, particularly for the *a posteriori* occupations and industries and for subgroup analyses. In addition, similar to many recent population-based studies, the response rates among the control subjects were not optimal. However, the sample weights included adjustments for differential nonresponse rates among demographic categories that may reduce bias in the analyses due to the nonresponse. The weights may also be useful in generalizing results to the Detroit and Chicago areas given that our control sources were approximately representative of the general population. Compared to the 2000 U.S. Census the population coverage rate for Chicago and Detroit for 20–64 year olds by the DMV records in 2002 (for Chicago) and 2003 (for Detroit) was about 100% for males and 96% for females. For Wayne County alone the coverage was lower, about 93% for males and 87.1% for females. We found the population coverage in Wayne County to be higher among 45–64 year olds, about 97% for males and 92% for females, which was the age range of most of the cases [49]. The sample sizes for many of the occupations and industries were small, leading to wide confidence intervals. While we had sufficient power to detect relatively small main effects, the power for stratified analysis by sex and race was limited. Finally, job titles are only surrogates for exposure. A specific job title may be associated with a wide range of different possible exposures; grouping subjects who may be highly exposed with those potentially unexposed mitigates the strength of the association.

## Conclusions

In summary, our findings from this large population-based case–control study of Caucasians and African Americans offer support for associations with RCC in the agricultural and dry-cleaning industries, and suggest for the first time that these associations might be stronger for the ccRCC subtype. These findings, along with suggestive associations observed for other occupations and industries, offer new leads worthy of further epidemiologic investigation.

## Abbreviations

AHS, Agricultural Health Study; BMI, Body mass index; ccRCC, Clear cell renal cell carcinoma; CI, Confidence interval; DMV, Department of Motor Vehicle; ICD-O, International Classification of Disease for Oncology; OR, Odds ratio; PCE, tetrachloroethylene; RCC, Renal cell carcinoma; SIC, Standard Industry Classification; SMR, Standardized mortality ratio; SOC, Standard Occupational Classification; TCE, Trichloroethylene; U.S., United States; USA, United States of America; VHL, Von Hippel-Lindau.

## Competing interests

The authors declare that they have no competing interests.

## Authors’ contributions

KS, FGD, JJR, NR and W-HC designed the study and collected the data. SK, JSC, SSM, SW, PAS, BIG and MPP analyzed the data. SK, JSC, and MPP drafted the manuscript. All authors read, gave comments, and approved the final version of the manuscript. SK had full access to all the data in the study and take responsibility for the integrity of the data and accuracy of the data analysis. All authors have read and approved the final manuscript.

## Pre-publication history

The pre-publication history for this paper can be accessed here:

http://www.biomedcentral.com/1471-2407/12/344/prepub

## Supplementary Material

Additional file 1 ** Table S1.** Weighted analysis for renal cell carcinoma risk by industry, by sex. Click here for file

Additional file 2 ** Table S2.** Occupation and risk of renal cell carcinoma, by sex. Click here for file
